# miR-494-3p Induces Cellular Senescence and Enhances Radiosensitivity in Human Oral Squamous Carcinoma Cells

**DOI:** 10.3390/ijms17071092

**Published:** 2016-07-08

**Authors:** Jui-Hung Weng, Cheng-Chia Yu, Yueh-Chun Lee, Cheng-Wei Lin, Wen-Wei Chang, Yu-Liang Kuo

**Affiliations:** 1 Department of Nuclear Medicine, Chung Shan Medical University Hospital, Taichung 40201, Taiwan; cshy695@csh.org.tw; 2 School of Dentistry, Chung Shan Medical University, Taichung 40201, Taiwan; ccyu@csmu.edu.tw; 3 Department of Dentistry, Chung Shan Medical University Hospital, Taichung 40201, Taiwan; 4 Institute of Oral Science, Chung Shan Medical University, Taichung 40201, Taiwan; 5 Department of Radiation Oncology, Chung Shan Medical University Hospital, Taichung 40201, Taiwan; lee.yuehchun@gmail.com; 6 Institute of Medicine, Chung Shan Medical University, Taichung 40201, Taiwan; 7 School of Biomedical Sciences, College of Medical Science and Technology, Chung Shan Medical University, Taichung 40201, Taiwan; lcu9281@gmail.com; 8 Department of Medical Research, Chung Shan Medical University Hospital, Taichung 40201, Taiwan; 9 Department of Medical Imaging and Radiological Sciences, Chung Shan Medical University, Taichung 40201, Taiwan; 10 Department of Medical Imaging, Chung Shan Medical University Hospital, Taichung 40201, Taiwan

**Keywords:** miR-494-3p, oral squamous cell carcinoma, radiotherapy, senescence, Bmi1

## Abstract

Oral squamous cell carcinoma (OSCC) is the most common malignancy of head and neck. Although radiotherapy is used for OSCC treatment, the occurrence of radioresistant cancer cells limits its efficiency. MicroRNAs (miRNAs) are non-coding RNAs with lengths of 18–25 base pairs and known to be involved in carcinogenesis. We previously demonstrated that by targeting B lymphoma Mo-MLV insertion region 1 homolog (Bmi1), miR-494-3p functions as a putative tumor suppressor miRNA in OSCC. In this study, we further discovered that miR-494-3p could enhance the radiosensitivity of SAS OSCC cells and induce cellular senescence. The overexpression of miR-494-3p in SAS cells increased the population of senescence-associated β-galactosidase positive cells, the expression of p16^INK4a^ and retinoblastoma 1 (RB1), as well as downregulated Bmi1. The knockdown of Bmi1 by lentiviral-mediated delivery of specific short hairpin RNAs (shRNAs) also enhanced the radiosensitivity of SAS cells and the activation of the senescence pathway. Furthermore, the inverse correlation between Bmi1 and miR-494-3p expression was observed among OSCC tissues. Results suggest that miR-494-3p could increase the radiosensitivity of OSCC cells through the induction of cellular senescence caused by the downregulation of Bmi1.

## 1. Introduction

Oral squamous cell carcinoma (OSCC) is the main type of cancer in the oral cavity; it presents poor prognosis with a five-year survival rate of less than 60% [1]. Radiotherapy is one of the current treatments for OSCC, but its therapeutic effect is limited by the development of radioresistance [2]. Enhancing radiosensitivity of cancer cells is one of the developing directions in cancer radiotherapy [3,4]. Cellular responses of radiation include cell cycle arrest, apoptosis, mitotic catastrophe, autophagy, and cellular senescence [5]. Cellular senescence is an irreversible cell cycle arrest and is considered as a potent mechanism of the suppressive effect of anti-cancer drugs [3,6]. The signaling transduction of cellular senescence includes p53-p21 and p16^INK4a^-retinoblatoma 1 (RB1) pathways [7]. p16^INK4a^ (also known as cyclin-dependent kinase inhibitor 2A, CDKN2A) can bind to CDK4/6, inhibiting its kinase activity and leading to the prevention of RB1 phosphorylation [8]. Unphosphorylated RB1 remains bound to the E2F transcription factor 1 (E2F1) and suppresses the expression of genes required for G1 to S transition [9].

MicroRNAs (miRNAs) are non-coding RNAs with a length of 18–25 base pairs and function as gene regulators by binding to the 3′-untranslated region (UTR) of the target mRNA, followed by mRNA degradation or inhibition of translation [10]. MicroRNAs are reportedly involved in numerous cellular processes, such as development, differentiation, proliferation, and apoptosis [11]. Several miRNAs are involved in the carcinogenesis of OSCC [12]. We previously demonstrated that the overexpression of miR-494-3p in head and neck cancer-derived tumor initiating cells (HNC-TICs) reduces cancer stemness through the downregulation of B lymphoma Mo-MLV insertion region 1 homolog (Bmi1) [13].

Previous studies reported that HNC-TICs displayed a radioresistant feature [14,15]. The present study further examined the function of miR-494-3p and its target gene Bmi1 in the radiation response of the SAS OSCC cell line. We showed that the overexpression of miR-494-3p or knockdown of Bmi1 enhanced the radiosensitivity in SAS cells and activated the cellular senescence pathway.

## 2. Results

### 2.1. miR-494-3p Enhanced Radiosensitivity in SAS OSCC Cells

**Figure 1:**
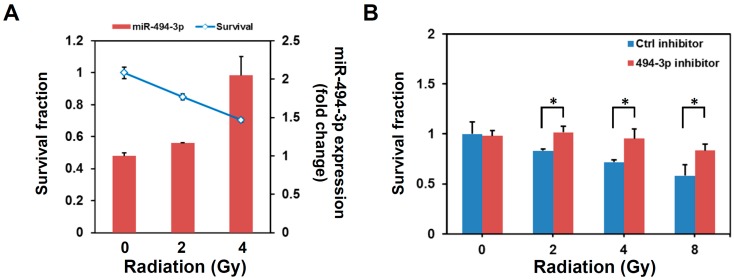

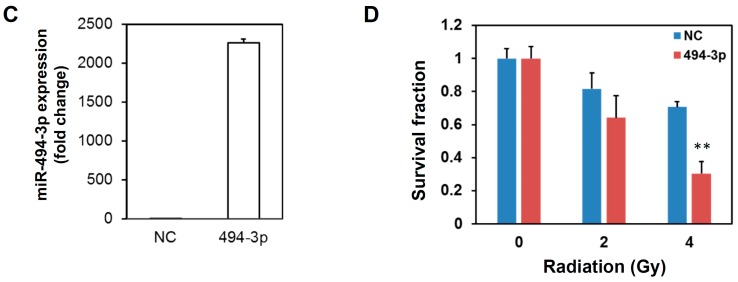
miR-494-3p regulates radiosensitivity of SAS oral squamous cell carcinoma (OSCC) cells. (**A**) SAS cells were irradiated as 2 or 4 Gy and cultured for further 72 h. Cell viability was determined by WST-1 reagent and miR-494-3p expression was measured by qRT-PCR; (**B**) SAS cells were transfected with 100 nM miR-494-3p inhibitor (494-3p inhibitor) or negative control inhibitor (Ctrl inhibitor) for 48 h. Cells were irradiated as 2, 4, or 8 Gy and cultured for further 72 h followed by determination of cell viability with WST-1. Data were presented as survival fraction as comparison with non-irradiated cells. *, *p* < 0.05; (**C**,**D**) SAS cells were transfected with 100 nM miR-494-3p mimic (494-3p) or negative control mimic (NC) for 48 h. The expression of miR-494-3p was determined by qPCR (**C**). Transfected SAS cells were irradiated as 2 or 4 Gy and cultured for further 72 h. Cell viability was determined by WST-1 reagent. Data were presented as survival fraction as comparison with non-irradiated cells (**D**). **, *p* < 0.01.

We previously discovered that the overexpression of miR-494-3p in aldehyde dehydrogenase 1 (ALDH1)+ CD44+ HNC-TICs inhibited their cancer stemness [13]. Given that radioresistance is one of the properties of HNC-TICs [15], the correlation between radiation response and miR-494-3p expression was examined first. As shown in Figure 1, the expression of miR-494-3p displayed a negative correlation with cell survival after radiation in SAS cells (Figure 1A). After transfection with the miR-494-3p inhibitor to inhibit the intracellular level of miR-494-3p, the SAS cells became resistant to radiation (Figure 1B). We next examined if the overexpression of miR-494-3p in the SAS OSCC cell line could enhance radiosensitivity. After the transient transfection of the miR-494-3p mimic in SAS cells, the miR-494-3p was increased more than two thousand-fold in comparison with a negative control mimic at 24 h post-transfection (Figure 1C). Overexpression of miR-494-3p in SAS cells displayed significantly enhanced sensitivity to an irradiation of 4 Gy (Figure 1D, *p* = 0.0001). These results indicate that miR-494-3p regulates the radiosensitivity of SAS OSCC cells.

### 2.2. miR-494-3p Induced Cellular Senescence in SAS Cells

**Figure 2:**
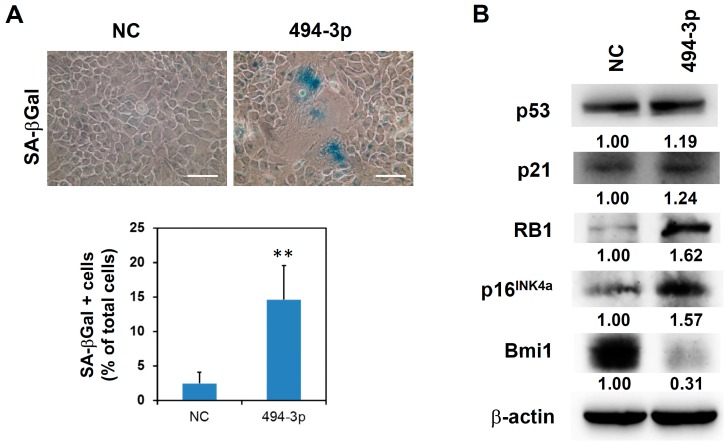
Overexpression of miR-494-3p induces cellular senescence in SAS cells. SAS cells were transfected with 100 nM miR-494-3p mimic (494-3p) or negative control mimic (NC). (**A**) Cellular senescence was determined by senescence-associated β-galactosidase (SA-β-Gal) staining at day 7 post-transfection. The quantification results were collected by 3 random fields of each miRNA mimic transfected samples. Scale bar: 50 μm. **, *p* < 0.01; (**B**) The expression of p53, p21, p16^INK4a^, retinoblastoma 1 (RB1), or B lymphoma Mo-MLV insertion region 1 homolog (Bmi1) was determined by Western blot at day 2 post-transfection. β-actin was used as an internal control. The inserted numbers indicated relative expression levels as comparison with NC group.

**Figure 3:**
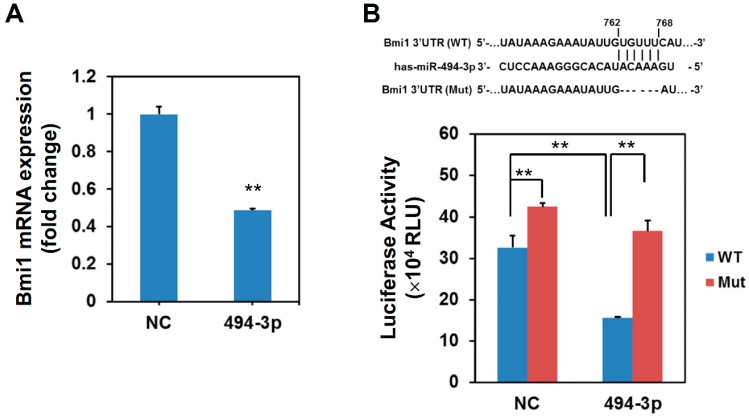
Bmi1 was a target of miR-494-3p in SAS cells. (**A**) Negative control mimic (NC) or miR-494-3p mimic (494-3p) was transfected into SAS cells and total RNA were extracted at 48 h post-transfection. The mRNA expression of Bmi1 was determined by qRT-PCR. **, *p* < 0.01; (**B**) Schematic presentation of the constructed Bmi1 3′-untranslated region (UTR) reporter plasmids were used in this study. WT, wild type; Mut, mutant. SAS cells were transfected with negative control mimic (NC) or miR-494-3p mimic (494-3p) simultaneously with Bmi1 3′-UTR reporter plasmid for 48 h. The cells were then lysed with passive lysis buffer and the luciferase activity was determined. **, *p* < 0.01.

Cellular senescence is one of the mechanisms in cellular pathways induced by radiation [5]. We next examined if the overexpression of miR-494-3p could induce cellular senescence in SAS cells. The detection of senescence-associated β-galactosidase (SA-β-Gal) activity indicated that transfection of the miR-494-3p mimic in SAS cells significantly induced cellular senescence at day 7 post-transfection (Figure 2A, 2.47% ± 1.61% in negative control mimic-transfected cells versus 14.63% ± 4.97% in miR-494-3p mimic-transfected cells, *p* = 0.0035). The expression of p16^INK4a^ and RB1 was obviously upregulated by the overexpression of miR-494-3p (Figure 2B), whereas that of p53 and p21 was only slightly increased by the overexpression of miR-494-3p (Figure 2B). We previously found that Bmi1 was one of the targets of miR-494-3p [13]. Here, we also confirmed that Bmi1 was inhibited by the overexpression of miR-494-3p mimic in SAS cells (Figure 2B). The overexpression of miR-494-3p in SAS cells also significantly inhibited the mRNA expression of Bmi1 (Figure 3A). Luciferase activity was inhibited when the luciferase gene contained full-length Bmi1 3′-UTR, whereas luciferase activity was not affected when the potential target site was deleted (Figure 3B).

### 2.3. Knockdown of Bmi1 Increased Radiosensitivity and Induced Senescence Pathway in SAS Cells

**Figure 4:**
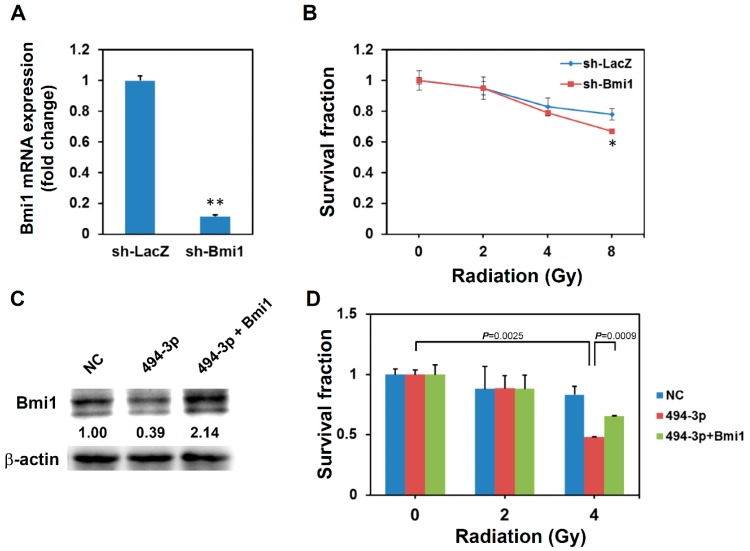
Knockdown of Bmi1 increases radiosensitivity in SAS cells. (**A**,**B**) SAS cells were transduced with sh-Bmi1 or sh-LacZ carrying lentivirus and selected with 2 μg/mL puromycin for 3 days. The knockdown efficiency was determined by qPCR detection of Bmi1 mRNA expression (**A**). **, *p* < 0.01; Transduced cells were irradiated as 2, 4, or 8 Gy and cultured for further 72 h. Cell viability was determined by WST-1 reagent. Data were presented as survival fraction as comparison with non-irradiated cells (**B**). *, *p* < 0.05; (**C**,**D**) SAS cells were transfected with pcDNA3-Bmi1 vector with miR-494-3p mimic (494-3p). A negative control mimic (NC) was used as control. After 48 h, cells were harvested for determination of the Bmi1 expression by Western blot (**C**) and the radiation responses of SAS cells (**D**).

**Figure 5:**
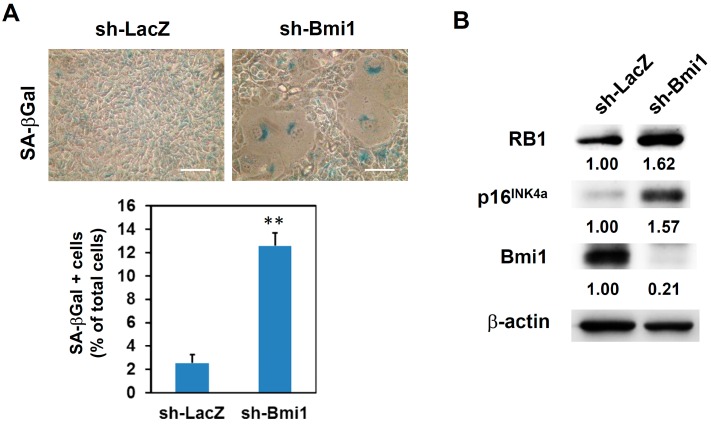
Knockdown of Bmi1 activates senescence pathway in SAS cells. SAS cells were transduced with sh-Bmi1 or sh-LacZ carrying lentivirus and selected with 2 μg/mL puromycin for 3 days. (**A**) sh-LacZ or sh-Bmi1 transduced SAS cells were harvested by trypsin/EDTA and seeded into 12-well-plate at a density of 2 × 10^4^ cells/well. The senescent cells were determined by SA-β-Gal staining at 72 h post seeding. Scale bar: 50 μm. **, *p* < 0.01; (**B**) The expression of p16^INK4a^, RB1, or Bmi1 was determined by Western blot. β-actin was used as an internal control. The inserted numbers indicate relative expression levels as compared with sh-LacZ group.

We hypothesized that the effect that miR-494-3p has on enhancing radiosensitivity in SAS cells is due to the downregulation of Bmi1. Thus, we used lentiviral-mediated short hairpin RNA (shRNA) delivery to knockdown Bmi1 expression in SAS cells and observed the radiation response of transduced cells. After the transduction of the Bmi1-specific shRNA lentivirus, the mRNA expression of Bmi1 was significantly inhibited (Figure 4A). The radiosensitivity of sh-Bmi1 lentivirus-transduced SAS cells was increased at the radiation dosage of 8 Gy (Figure 4B). The overexpression of Bmi1 with a pcDNA3-Bmi1 vector (Figure 4C) in SAS cells displayed a partial recovery of radiosensitization effect of the miR-494-3p mimic (Figure 4D). We next examined whether the knockdown of Bmi1 in SAS cells could activate cellular senescence pathway. SA-β-Gal staining revealed that the knockdown of Bmi1 in SAS cells significantly increased the percentage of senescent cells (Figure 5A). As seen using Western blot analysis, the expression levels of p16^INK4a^ and RB1 were increased in sh-Bmi1 lentivirus-transduced SAS cells (Figure 5B). These results suggested that miR-494-3p could enhance radiosenesivity in SAS cells through the induction of cellular senescence caused by the downregulation of Bmi1.

### 2.4. miR-494-3p Was Inversely Correlated with Bmi1 Expression among OSCC Patients

**Figure 6:**
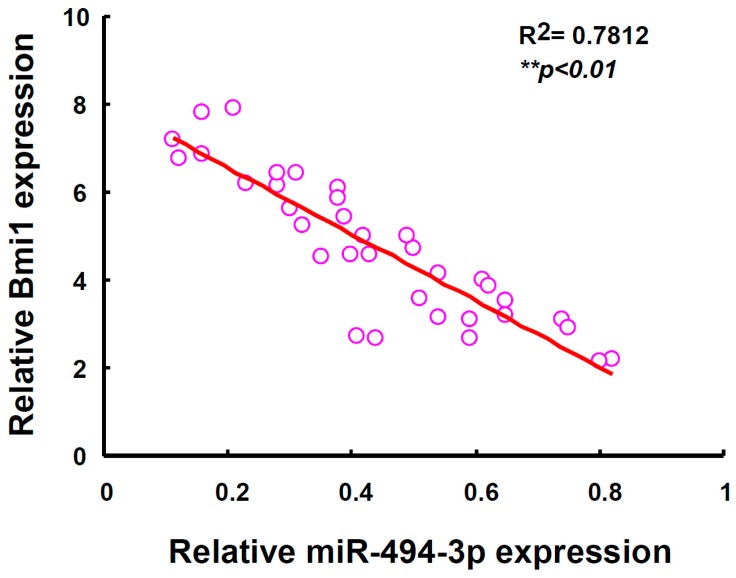
The negative correlation between miR-494-3p and Bmi1 expression in HNC patients. Total RNA were extracted from tumor tissues of head and neck cancer (HNC) patients (*n* = 35) and the expression of Bmi1 and miR-494-3p was determined by qRT-PCR methods and analyzed with Spearman rank correlation test.

We previously discovered that miR-494-3p expression was significantly decreased in stage III or stage IV OSCC patients [13]. OSCC patients with low miR-494-3p or high Bmi1 expression displayed poor survival rates [13]. We further analyzed the expression of miR-494-3p and Bmi1 among OSCC patients, and the results revealed a significantly inverse correlation between miR-494-3p and Bmi1 expression among OSCC patients (Figure 6).

## 3. Discussion

The tumor suppression effect of miR-494-3p has been reported in pancreatic cancer [16], cervical cancer [17], small cell lung cancer [18], and OSCC [13,19]. By contrast, the oncogenic role of miR-494-3p has been shown in glioma [20], non-small cell lung cancer [21] and hepatocellular carcinoma [22]. The role of miR-494-3p in carcinogenesis appears to be dependent on cancer types. The present study demonstrated that miR-494-3p induced cellular senescence and downregulation of Bmi1 in SAS OSCC cells (Figure 2). These data further support the tumor-suppressing function of miR-494-3p in OSCC.

Ohdaira et al. previously found that the overexpression of miR-494-3p in A549 lung cancer cells induced cellular senescence through inhibition of insulin-like growth factor 2 mRNA-binding protein 1 [23]. Comegna et al. also demonstrated that the overexpression of miR-494-3p in human diploid IMR90 fibroblasts led to senescence through the downregulation of heterogeneous nuclear ribonucleoprotein A3 and UV excision repair protein RAD23 homolog B [24]. In our study, the overexpression of miR-494-3 displayed greater efficiency in enhancing radiosensitivity in SAS cells than the knockdown of Bmi1. The growth inhibition was significantly increased in the miR-494-3p overexpression group receiving 4 Gy irradiation (Figure 1B), but the enhanced radiosensitivity in Bmi1 knockdown cells was only observed when these were irradiated at 8 Gy (Figure 4B). This difference indicated that other target genes may also be involved in the induction of cellular senescence by miR-494-3p in SAS OSCC cells. We observed that the overexpression of miR-494-3p not only suppressed the expression of Bmi1 (Figure 2B and Figure 3A) but also downregulated c-Myc mRNA (Figure S1). However, the knockdown of Bmi1 slightly increased the expression of c-Myc (Figure S2). c-Myc is one of the targets of miR-494-3p [16,25] and is essential for the inhibition of senescence mediated by CDK2 [26]. On the other hand, other targets of miR-494-3p may also be involved in the radiosensitization effect of miR-494-3p in OSCC cells. HOXA1, a member of homeobox genes superfamily, has been reported as a target of miR-494-3p in oral cancer [19]. HOXA1 has also been reported to participate in temozolomide resistance in glioblastoma cell lines through the regulation of the homologous recombinant DNA repair pathway [27]. Determining whether HOXA1 is also involved in the radiosensitization effect of miR-494-3p in OSCC cells requires further studies. The simultaneous inhibition of Bmi1 and c-Myc, as well as other potential target genes, by overexpression of miR-494-3p may explain the better efficiency in enhancing radiosensitivity in SAS cells; however, this finding remains to be further investigated.

## 4. Materials and Methods

### 4.1. Cell Culture and the Determination of Radiosensitivity

SAS cells were maintained in Dulbecco modified Eagle’s medium (DMEM) (Gibco, Invitrogen Corporation, Carlsbad, CA, USA) containing 10% fetal bovine serum (FBS) (Gibco) in a standard humidified incubator at 37 °C in 5% CO_2_ . For determination of radiosensitivity, cells were suspended as 1 × 10^6^ cells/mL in DMEM medium containing 10% FBS and 1.5 mL microtubes and irradiated by Elekta Axesse linear accelerator (Elekta AB, Stockholm, Sweden) at a dose rate of 6 Gy·min^−1^. Irradiated cells were then seeded into wells of 96-well plates at a density of 1 × 10^3^/well and cultured for 72 h. Cell viability was determined by WST-1 reagent (Roche Life Science, Indianapolis, IN, USA).

### 4.2. Transfection of miRNA Mimic, Bmi1 3′UTR Reporter or pcDNA3-Bmi1 Plasmids

The miR-494-3p mimic and negative control mimic were purchased from RiboBio Co., Ltd. (Guangzhou, China). Cells were seeded in wells of 12-well-plate as 1 × 10^5^ cells/well for attachment overnight and miRNA mimic was transfected by Lipofectamine^TM^ 3000 transfection reagent (Invitrogen, Thermo Fisher Scientific Inc., Carlsbad, CA, USA) at a concentration of 100 nM according to the manufacturer’s protocol. A firefly luciferase reporter plasmid with full length Bmi1 3′-UTR sequence was purchased from OriGene Technologies, Inc. (Rockville, MD, USA). The mutant Bmi1 3′-UTR reporter plasmid used for deletion of the potential miR-494-3p binding region (Figure 3A) was further constructed by QuickChange II XL Site-Directed Mutagenesis Kit (Agilent Technologies Inc., Santa Clara, CA, USA) with following primers: 5′-gggagaattttaacaatcatttctgaatgcatcaatatttctttatagcatttataaatatatc-3′ and 5′-gatatatttataaatgctataaagaaatattgatgcattcagaaatgattgttaaaattctccc-3′. For dual luciferase activity assay, 100 nM miRNA mimic was simultaneously transfected with 1 μg of Bmi1 3′-UTR reporter plasmid and 0.1 μg pRL-TK plasmid (Promega Corporation, Madison, MI, USA) by Lipofectamine^TM^ 3000 transfection reagent. Cells were lysed at 48 h post-transfection by passive lysis buffer (Promega) and the activity of firefly and renilla was determined with Dual-Luciferase^®^ Reporter System (Promega). The pCDNA3-Bmi1 plasmid was a gift from Professor Muh-Hwa Yang (Institute of Clinical Medicine, National Yang-Ming University, Taiwan) and 1 μg/well was used for transfection in 6-well plate. After transfection for 48 h, cells were harvested for radiation response assay as described in Section 4.1.

### 4.3. Detection of Senescence Associated β-Galactosidase Activity

Cellular senescence was determined by measuring the activity of SA-β-Gal with a Cellular Senescence Assay kit purchased from Merck Millipore (Darmstadt, Germany). Briefly, cells were fixed with the fixation buffer at room temperature for 15 min and washed with phosphate-balanced saline. The SA-β-Gal activity was then detected by detection solution at 37 °C without CO_2_ for 4 h. The blue stained cells, which represented SA-β-Gal positive cells, were pictured and counted under an inverted light microscopy (AE30, Motic Electric Group Co., Ltd., Xiamen, China).

### 4.4. Western Blot Analysis

Cells were harvested by trypsin/EDTA and lysed in M-PER Mammalian Protein Extraction Reagent (Pirece Thermo Fisher Scientific Inc., Waltham, MA, USA). Twenty micrograms of total protein were separated by sodium dodecyl sulfate (SDS)-polyacrylamide gel electrophoresis (PAGE) and transferred onto polyvinylidene difluoride (PVDF) membrane (Immobilon-P, Merck Millipore). The membrane was then blocked with 5% skimmed milk (Sigma-Aldrich, St. Louis, MO, USA) dissolved in Tris-buffered sline (TBS)(Sigma-Aldrich) containing 0.05% Tween-20 (Sigma-Aldrich) (TBS-T) at room temperature for 1 h followed by incubation with primary antibodies at 4 °C overnight (rabbit anti-human Bmi1, p16^INK4a^, or RB1 polyclonal antibodies were purchased from Novus Biologicals, LLC, Littleton, CO, USA; polyclonal rabbit anti-human p53 antibody or monoclonal mouse anti-human p21 antibody were purchased from GeneTex International Corporation, Hsinchu City, Taiwan; mouse anti-human β-actin monoclonal antibody was purchased from Sigma-Aldrich; mouse anti-flag monoclonal antibody was purchased from Proteintech Group, Rosemont, IL, USA). After washing with TBS-T, the membrane was then incubated with peroxidase-conjugated secondary antibodies (PerkinElmer, Waltham, MA, USA) at room temperature for 1 h. The signals were developed by ECL-plus chemiluminescence substrate (PerkinElmer) and captured using a Luminescent Image Analyzer (Fusion SOLO, Vilber Lourmat, Marne-la-Vallée, France). The band intensity was quantified using ImageJ software (NIH, Bethesda, MA, USA).

### 4.5. qPCR

Total RNA was extracted by Quick-RNA^TM^ MiniPrep Kit (Zymo Research Corporation, Irvine, CA, USA). For miR-494-3p detection, 100 ng extracted RNA was used for complementary DNA (cDNA) synthesis with RevertAid First Strand cDNA Synthesis Kit (Thermo Fisher Scientific Inc.) and a specific RT primer (RiboBio Co., Ltd.). qPCR was performed on an ABI StepOnePlus™ Real-Time PCR System (Applied Biosystems, Life Technologies Corp., Carlsbad, CA, USA) by SYBR Green reagent (SYBR^®^ FAST qPCR Kit, Kapa Biosystems, Inc., Wilmington, MA, USA) with specific primers purchased from RiboBio Co., Ltd. and analyzed with the StepOne software (Applied Biosystems). For Bmi1 detection, 1 μg of extracted RNA was used for cDNA synthesis with random hexamers provided in RevertAid First Strand cDNA Synthesis Kit followed by SYBR Green based qPCR reaction. The primer sequences were listed as followed:

Bmi1
Forward: 5′-AATCCCCACCTGATGTGTGT-3′
Reverse: 5′-GCTGGTCTCCAGGTAACGAA-3′
MRPL19 (internal control)
Forward: 5′-GGGATTTGCATTCAGAGATCAG-3′
Reverse: 5′-GGAAGGGCATCTCGTAAG-3′
qPCR data were analyzed as previous described [28].

### 4.6. Lentivirus-Based shRNA Delivery

Bmi1-specific shRNA plasmids (TRCN0000229416 and TRCN0000218869) or sh-LacZ lentivirus virus solution were obtained from the National RNAi Core Facility at the Institute of Molecular Biology (Academia Sinica, Taipei, Taiwan). The production of sh-Bmi1 carrying lentivirus was performed by co-transfection of sh-Bmi1/pCMV-ΔR8.91/pMD.G plasmids as a ratio of 1:0.9:0.1 into 293T cells. The sh-Bmi1 viruses were mixed as 1:1 and transduced into SAS cells (MOI = 1) in presence of 8 μg/mL polybrene (Sigma-Aldrich) at 37 °C for 24 h. Transduced cells were selected by 2 μg/mL puromycin at 37 °C for 3 days and survived cells were harvested for further experiments.

### 4.7. Clinical OSCC Sample Analysis

The acquirement and preparation of OSCC tissues was approved by the institutional review board of Chung Shan Medical University. The procedures of resected tissues from OSCC patients were as previously described [13]. The correlation between Bmi1 and miR-494 in tumor tissues was analyzed by real-time RT-PCR analysis and Spearman rank correlation test.

## 5. Conclusions

We discovered that the overexpression of miR-494-3p in SAS OSCC cells could induce cellular senescence and enhance radiosensitivity, effects that may be mediated by the downregulation of Bmi1. Furthermore, an inverse correlation was observed between miR-494-3p and Bmi1 expression among OSCC patients. Compounds that are actively involved in the induction of miR-494-3p expression, including silibinin [13], could be considered as potential radiation sensitization agents for OSCC therapy.

## Supplementary Materials

Supplementary materials can be found at http://www.mdpi.com/1422-0067/17/7/1092/s1.
