# Supplementary material for: miR-494-3p Induces Cellular Senescence and Enhances Radiosensitivity in Human Oral Squamous Carcinoma Cells
Source: Int J Mol Sci. 2016 Jul 8;17(7):1092. doi: 10.3390/ijms17071092 (PMC4964468; doi:10.3390/ijms17071092)
Supplement: Supplementary file 1 [file ijms-17-01092-s001.pdf]

Supplementary Materials: miR-494-3p Induces Cellular Senescence and Enhances Radiosensitivity in Human Oral Squamous Carcinoma Cells

Jui-Hung Weng, Cheng-Chia Yu, Yueh-Chun Lee, Cheng-Wei Lin, Wen-Wei Chang and Yu-Liang Kuo

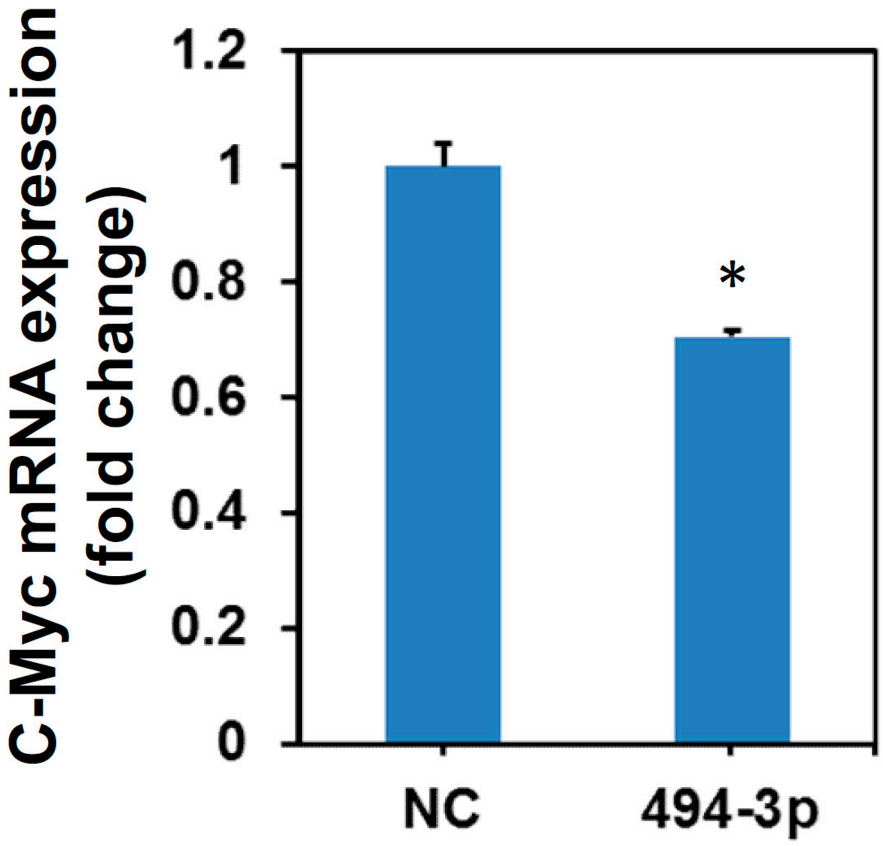

Figure S1. Overexpression of miR-494-3p downregulates c-Myc in SAS cells. Cells were transfected with 100 nM of negative control mimic (NC) or miR-494-3p mimic (494-3p) for 48 h. The expression of c-Myc was determined by qRT-PCR. *, $p < 0.05$; **, $p < 0.01$.

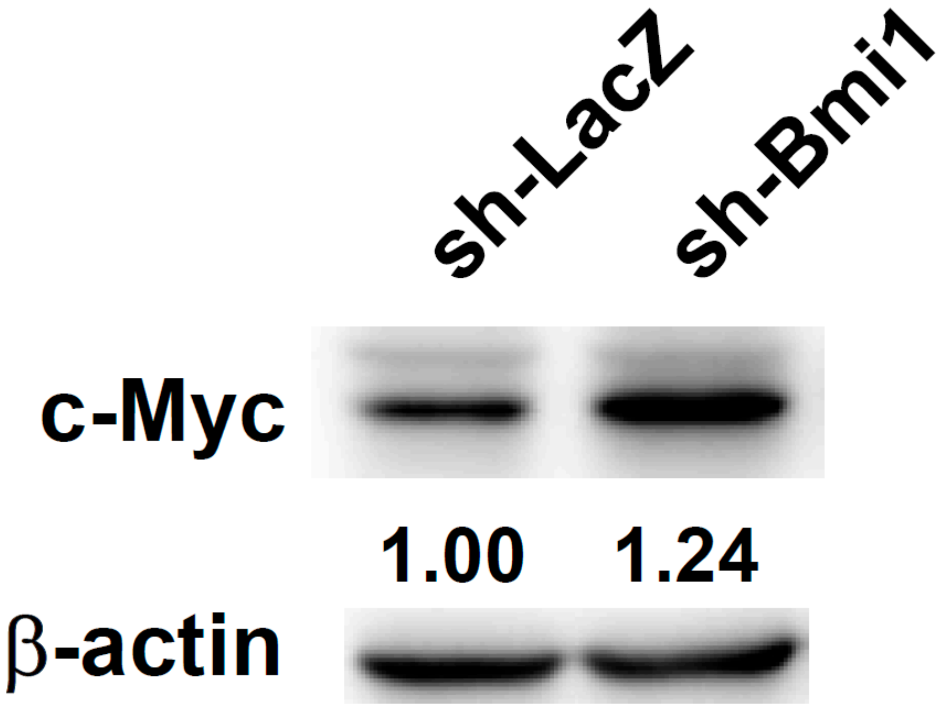

Figure S2. Knockdown of Bmi1 in SAS cells slightly increases c-Myc expression. Cells were transduced with lentivirus carrying sh-LacZ or sh-Bmi1 and selected with 2 μg/mL puromycin for 3 days. The expression of c-Myc was determined by Western blot. The inserted numbers indicated relative expression levels as comparison with sh-LacZ group
